# L-Carnitine in Drosophila: A Review

**DOI:** 10.3390/antiox9121310

**Published:** 2020-12-21

**Authors:** Maria Rosaria Carillo, Carla Bertapelle, Filippo Scialò, Mario Siervo, Gianrico Spagnuolo, Michele Simeone, Gianfranco Peluso, Filomena Anna Digilio

**Affiliations:** 1 Elleva Pharma S.R.L. via P. Castellino, 111, 80131 Naples, Italy; mariarosaria.carillo@unicampania.it; 2 Department of Neurosciences, Reproductive and Odontostomatological Sciences, University of Naples “Federico II”, Via Pansini 5, 80131 Naples, Italy; carla.bertapelle@unina.it (C.B.); gspagnuo@unina.it (G.S.); michele.simeone@unina.it (M.S.); 3 Dipartimento di Scienze Mediche Traslazionali, University of Campania “Luigi Vanvitelli”, 80131 Naples, Italy; filippo.scialo@unicampania.it; 4 Queen’s Medical Center, School of Life Sciences, The University of Nottingham Medical School, Nottingham NG7 2UH, UK; Mario.Siervo@nottingham.ac.uk; 5 Research Institute on Terrestrial Ecosystems (IRET)-CNR, Via Pietro Castellino 111, 80131 Naples, Italy; gianfranco.peluso@cnr.it

**Keywords:** *Drosophilamelanogaster*, L-carnitine, energy metabolism, neurodegenerative diseases

## Abstract

L-Carnitine is an amino acid derivative that plays a key role in the metabolism of fatty acids, including the shuttling of long-chain fatty acyl CoA to fuel mitochondrial β-oxidation. In addition, L-carnitine reduces oxidative damage and plays an essential role in the maintenance of cellular energy homeostasis. L-carnitine also plays an essential role in the control of cerebral functions, and the aberrant regulation of genes involved in carnitine biosynthesis and mitochondrial carnitine transport in Drosophila models has been linked to neurodegeneration. Drosophila models of neurodegenerative diseases provide a powerful platform to both unravel the molecular pathways that contribute to neurodegeneration and identify potential therapeutic targets. Drosophila can biosynthesize L-carnitine, and its carnitine transport system is similar to the human transport system; moreover, evidence from a defective Drosophila mutant for one of the carnitine shuttle genes supports the hypothesis of the occurrence of β-oxidation in glial cells. Hence, Drosophila models could advance the understanding of the links between L-carnitine and the development of neurodegenerative disorders. This review summarizes the current knowledge on L-carnitine in Drosophila and discusses the role of the L-carnitine pathway in fly models of neurodegeneration.

## 1. Introduction

Biological processes involved in the regulation of energy metabolism are highly conserved in all living organisms. Carbohydrates, amino acids, and lipids are the primary substrates utilized by animals to generate energy in the form of ATP. L-Carnitine plays a key role in lipid metabolism by transporting fatty acids into the mitochondria of the cell where they are converted into energy. L-Carnitine is also known as vitamin T and is a small water-soluble zwitterion classified as a quaternary ammonium compound.

L-Carnitine acts as a cofactor for carnitine acyltransferases, a family of enzymes that catalyzes the exchange of acyl groups between L-carnitine and coenzyme A (CoA), to form acylcarnitines [1]. Carnitine acyltransferases are grouped in different classes based on their specificity for the length of the fatty acyl group used as a substrate: (1) Carnitine acetyltransferase (CrAT or CAT) uses acetyl-CoA as a substrate [2]; (2) Carnitine octanoyltransferase (CrOT or COT) regulates the peroxisomal metabolism of Very-Long-Chain Fatty Acids (VLCFA) and branched-chain fatty acids by promoting the transport of medium-length acyl (C8–C10) from peroxisomes to mitochondria for further breakdown [3,4]; (3) Carnitine palmitoyltransferase (CPTs) 1 and 2 are enzymes located on the outer and inner mitochondrial membrane, respectively, and are responsible for delivering Long-Chain Fatty Acids (LCFA) (C16–C20) into the mitochondrial matrix where they enter β-oxidation [1,5].

The primary function of L-carnitine is linked to the regulation of the mitochondrial metabolism of LCFA, as β-oxidation represents an important source of energy during fasting periods or intense physical activity. Whereas medium- or short-chain fatty acids can be directly imported into the mitochondria, LCFA cannot enter into the mitochondria by simple diffusion as they require a transport system known as Carnitine Shuttle (CS). This system is composed of three proteins: two different Carnitine Palmitoyltransferases, CPT1 and CPT2, and the Carnitine Acyl-Carnitine Translocase (CACT), which acts as a transporter; altogether, these proteins allow the transfer of activated FA into the mitochondrial matrix [5,6,7,8]. L-Carnitine is also involved in several biochemical pathways; specifically, it modulates the acyl-CoA/free CoA ratio [2,5,9] and has a role in shuttling chain-shortened medium- and short-chain acyl-CoAs groups from the peroxisomal matrix into the mitochondria to undergo further oxidation [10,11,12]. In addition, L-carnitine is involved in energy storage in the form of acetyl-carnitine [9,13] and binds partially metabolized, toxic acyl groups to allow their elimination [8,9,14,15,16,17,18]. L-Carnitine is associated with other biological processes, including (1) improved insulin sensitivity [17,19], (2) the acetylation of histones [20,21], and (3) anti-inflammatory and (4) antioxidant processes [22,23,24,25]. It is therefore not surprising that alterations in L-carnitine metabolism can be associated with the onset of different disorders such as myopathy, cardiomyopathy, hypoglycemia, fatty liver, and male infertility [26].

β-oxidation also plays a key role in cancer cells as these cells are dependent on lipid oxidation for their growth and proliferation [27]; in addition, several types of cancer present a dysregulation in the expression of both CPT1 and SLC22A5/OCTN2, which transports L-carnitine into the cells; therefore, these proteins have been considered a potential target for antineoplastic drugs [28].

β-oxidation has an important role in the regulation of brain function as its impairment may lead to neuropathy, Reye-like syndrome, seizures, mental retardation, and autism [29,30,31]. Lamhonwah et al. [32] recently reported an association between a deletion in the SLC22A5 gene with attention-deficit/hyperactivity disorder, and Celestino-Soper et al. [33,34] identified abnormal L-carnitine biosynthesis in several patients with autism spectrum disorders (ASD), suggesting that L-carnitine deficiency could represent a risk factor for autism and supporting the potential, beneficial effects of L-carnitine supplementation in these patients [35]. Dysregulation of the CS enzymes has been described in a Drosophila model of Amyotrophic Lateral Sclerosis (ALS) [36], and Laranjeira et al. [37] reported that defects in L-carnitine biosynthesis were responsible for aging-mediated brain decline. Recently, Di Cristo et al. [38,39] showed that pharmacological inhibition of the carnitine system in a Drosophila model of Huntington’s Disease (HD) leads to ameliorative effects on disease symptoms. The association of impaired L-carnitine metabolism with neurological pathologies and autism could advance the understanding of the pathophysiology of these disorders and identify novel therapeutic targets.

The key role played by L-carnitine in energy production is highly conserved across organisms; therefore, the study of the mechanisms linking β-oxidation and L-carnitine to neurodegenerative disorders could be facilitated in simpler animal models, such as the fruit fly *Drosophila melanogaster* [40]. Drosophila is a powerful model for studying human pathologies and neurodegenerations; it not only has a huge number of genetics and experimental tools but also has the added advantage of a less complex genome and less gene redundancy than mammals. At the same time, it maintains a high degree of genetic and metabolic similarities with humans [41], as about 70% of the genes responsible for human diseases have their counterparts in the fly [42]. Several disease models have been generated in Drosophila, the phenotypes of which recapitulate the key symptoms of the corresponding human disease [43,44]. The roles played by L-carnitine in brain physiology in Drosophila models are partially known, and this review aims to provide a critical appraisal of L-carnitine metabolism in Drosophila models and its relevance to the pathogenesis of human diseases. First, we will discuss the role of genes involved in the L-carnitine-biosynthesis pathway. Second, we will discuss what is known about L-carnitine genes regulating the shuttling of fatty acids in mitochondria and peroxisomes. Finally, the implications of L-carnitine pathway dysfunction in some neurodegenerative models will be discussed.

## 2. L-Carnitine Biosynthesis

In most animals, L-carnitine is obtained from the diet and synthesized endogenously from essential amino acids lysine and methionine. Protein-bound lysine contributes with the carbon backbone and methionine with the 4-N-methyl groups [45,46,47] to produce a N6-N6-N6-TriMethyl-l-Lysine (TML) form. This is the first metabolite of carnitine biosynthesis and is released in cells after lysosomal protein degradation [48,49,50,51,52].

**Figure 1:**
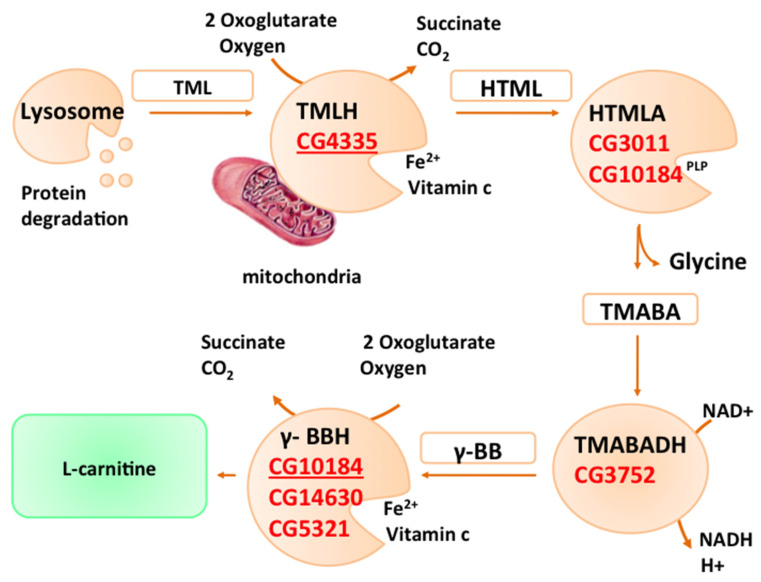
L-Carnitine biosynthesis pathway. Drosophila orthologs are reported near each counterpart with the FlyBase CG number(s) in red. The CG numbers with the best hit(s) are underlined.

At least four enzymes are involved in this biosynthetic pathway. In brief, as reported in Figure 1, TML is first hydroxylated by TML Hydroxylase (TMLH) to produce 3-Hydroxy-TriMethylLysine (HTML), which in turn is cleaved by β-Hydroxy-ε-N-TriMethylLysine Aldolase (HTMLA) into γ-TriMethylAminoButyrAldehyde (TMABA) and glycine. The resulting aldehyde is oxidized by γ-TriMethylAminoButyrAldehyde DeHydrogenase (TMABADH) to yield γ-ButyroBetaine (γBB), whose subsequent hydroxylation, catalyzed by γ-ButyoBetaine Hydroxylase (γ-BBH), generates L-carnitine [18,53,54].

Although the chemical characterization of the L-carnitine biosynthesis pathway was clarified more than 40 years ago, the identification and characterization of the corresponding genes were obtained later and not for all the enzymes involved [53,55,56,57,58].

To date, a functional carnitine biosynthesis pathway has not been described for *D. melanogaster*; however, the presence of putative orthologs of the carnitine biosynthesis genes in its genome lets us assume that, like humans, Drosophila can biosynthesize L-carnitine. The four genes of the L-carnitine pathway, TMLH, HTMLA, TMABADH, and γ-BBH, and their Drosophila orthologs are reported below.

### 2.1. TMLH

The first enzyme is TMLH, which catalyzes the hydroxylation reaction of TML to yield 3-hydroxy-TML (HTML). Genes encoding TMLH were identified [53,58] in humans, rats, mice, and the yeast *Candida albicans* and, recently, mutations of the human ortholog have been correlated to autistic disorder [36]. Blast analysis indicates that in Drosophila, *Dmel/CG4335* is the putative orthologous gene, which is an uncharacterized gene harbored on the right arm of chromosome 3. The coded protein shares a 55% of similarity with hTMLDE (trimethyllysine hydroxylase, epsilon) and has a consensus sequence for mitochondrion localization.

### 2.2. HTMLA

The second enzyme is β-hydroxy-ε-N-trimethyllysine aldolase (HTMLA), a pyridoxal phosphate-dependent aldolase, which catalyzes an aldolytic cleavage of HTML, generating γ-TriMethylAminoButyrAldehyde (TMABA) and glycine. The identity of its corresponding gene has remained elusive. Based on the reaction mechanism, it has been speculated that this enzyme could belong to Serine HydroxyMethylTransferase (SHMT) or Threonine Aldolase classes. The first genes encoding for HTMLA were identified in the yeast *C. albicans*, and correspond to two Threonine Aldolase genes (*orf19.6306* and *gly1*); however, genetic data suggest that these genes are necessary but not sufficient for this step and that a cytosolic SHMT may also be involved [58,59].

Besides *C. albicans*, to date, no other HTMLA-encoding gene has been cloned in any other organism. The closest Threonine Aldolase orthologs in mice and humans, known as *GLY1* and *THA1P,* respectively, are single-copy genes that have not yet been characterized at the molecular level. Although *GLY1* is a functional gene in rats and mice [60] and can be considered the functional gene encoding for HTMLA, *THA1P* in humans is reported as a pseudogene. Therefore, to explain the enzymatic HTMLA activity detected in different human tissues [61], it has been proposed that the cleavage reaction, which converts HTML into TMABA and glycine, is catalyzed by an enzyme such as SHMT with low HTMLA activity [62,63]. Drosophila has a single *SHMT* gene, referred to in FlyBase (http://flybase.org) by the symbol *DmelCG3011*, encoding the orthologs of the cytosolic isoform and the uncharacterized *DmelCG10184* gene that is classified as the ortholog of *Mmus/Tha1/GLY1* (*threonine aldolase 1*). However, there is no experimental evidence that these genes participate in the L-carnitine biosynthetic pathway.

### 2.3. TMABADH

The third enzyme of the carnitine biosynthesis pathway is the NAD^+^-dependent γ-TriMethylAminoButyrAldehyde DeHydrogenase (TMABADH), which catalyzes the dehydrogenation of TMABA into γ-ButyroBetaine (γ-BB) [64]. The corresponding gene has been identified in rats and other organisms [56]. Its promising human ortholog is the *aldehyde dehydrogenase 9 (ALDH9*) gene, based on a high degree of amino-acidic identity and highly similar substrate specificities with rat TMABADH [18,65,66]. To date, neither of the two orthologs have been identified in Drosophila, although one potential candidate is reported in FlyBase as *DmelCG3752*.

### 2.4. γ-BBH H

The fourth enzyme of the L-carnitine biosynthesis pathway is γ-ButyroBetaine hydroxylase (γ-BBH), which catalyzes the hydroxylation of γ-ButyroBetaine to yield L-carnitine [67]. It is considered the limiting step of the pathway, and γ-BBH is the most studied enzyme in this process. Its gene has been identified and characterized by several organisms, including humans [18,68].

In the Drosophila genome, there are three paralogous genes (*CG14630, CG5321, CG10184*) that are considered putative orthologs for the human γ-*BBH1* (γ-ButyroBetaine Hydroxylase 1) gene. In a recent study that aimed to identify genes contributing to the functional decline of the Drosophila brain during aging, Laranjeira et al. [39] suggested that the *CG10814* gene was the most likely ortholog for the *h*γ-*BBH1* gene, sharing with this the lack of a mitochondrial targeting sequence. Interestingly, these authors reported that *CG10814* transcription levels increased drastically with age and this upregulation had a negative impact on the functional decline of the brain with age; at the same time, its downregulation led to an improvement of neuronal activity. Taken together, their results suggest that in Drosophila, mitochondrial β-oxidation genes play an important role in defining the brain-aging phenotype.

## 3. L-Carnitine and Fatty Acid Oxidation

It is well known that L-carnitine plays an essential role in energy production through FA β-oxidation in maintaining normal mitochondrial function through the elimination of toxic products of the fatty acyl-CoA metabolism and in the modulation of the cellular acyl-CoA/CoASH ratio [2,8,14,69,70]. Fatty acid oxidation (FAO) takes place in two cellular compartments: peroxisomes and mitochondria, which are both involved in lipid homeostasis [71,72]. However, the oxidation that occurs in these two compartments is very different in terms of specificities and the transport of substrates, the end products, and energy yielding [71]. Long-, medium-, and short-chain fatty acids are oxidized inside the mitochondria in the process of β-oxidation [8]. VLCFA and branched- and medium-chain fatty acids undergo an incomplete β-oxidation in peroxisomes, and the final products are shuttled into the mitochondria for completing oxidation [73,74,75].

### 3.1. L-Carnitine and Mitochondria

**Figure 2:**
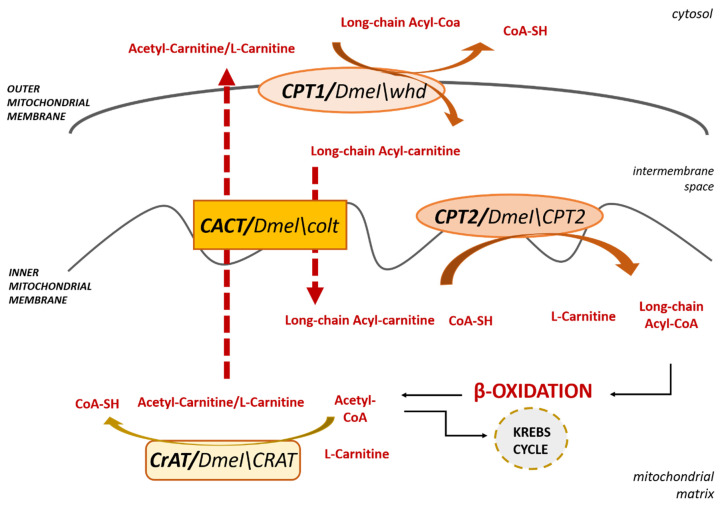
An overview of the Carnitine Shuttle. The Drosophila orthologs are reported near each enzyme with their *Dmel* name.

In order to enter the oxidative pathway into the mitochondria, intracellular FAs need to be activated to acyl-CoAs through a thioesterification reaction catalyzed by acyl-CoA synthases (ACSs) [76] and enter into the organelle matrix, the site of β-oxidation. Although medium- and short-chain fatty acids can cross mitochondrial membranes via diffusion, long-chain acyl-CoAs do not penetrate the lipid bilayer and require the CS transport system for mitochondrial access. As mentioned in Section 1, this transport system consists of both enzymes that reversibly transfer the acyl group from acyl-CoA to carnitine and carrier(s) that transport acyl moieties across the inner mitochondrial membrane. As shown in Figure 2, CPT1, localized in the outer mitochondrial membrane, transfers the acyl moiety of long-chain acylCoA to the hydroxyl group of L-carnitine to form acylcarnitine; then, CACT, an integral inner membrane protein, transfers the acylcarnitine across the inner plasma membrane through an exchange reaction with free L-carnitine/acetylcarnitines exiting the mitochondrial matrix. As a third step, CPT2, localized on the inner mitochondrial membrane, converts acylcarnitine back to free L-carnitine and acyl-CoA, which enters the β-oxidation cycle in the mitochondrial matrix, to produce acetyl-CoAs [77,78].

Finally, to close the carnitine cycle, CrAT, which resides in the matrix, reversibly transfers mitochondrial L-carnitine to medium- and short-chain acyl-CoA [2,79], allowing the export of the produced acetyl-CoA as acetyl-carnitine to the cytosol.

In Drosophila, this transport system is substantially similar to that occurring in humans. Below we report the Drosophila genes in comparison with human orthologs.

#### 3.1.1. Carnitine PalmitoylTransferase 1

CPT1 is an integral transmembrane protein of the mitochondrial outer membrane and catalyzes the first step of the CS, allowing the conversion of long-chain acyl-CoAs to their acylcarnitine equivalents. This enzyme can be strongly inhibited by malonyl-CoA, the first intermediate in FA synthesis, allowing a control switch between FA catabolism and synthesis. Humans, as well as other mammals, have three genes encoding three different isoforms of CPT1, specifically CPT1A, CPT1B, and CPT1C [14]. These isoforms differ in their tissue-specific expression, affinity for their substrate L-carnitine, and malonyl-CoA inhibition. CPT1A and CPT1B, also known as Liver-CPT1 and Muscle-CPT1, respectively, are the most highly expressed and widely studied isoforms [5,80,81]. The CPT1C isoform is the most recently discovered [82]. It was found in different phyla such as teleosts, amphibia, reptiles, lobe-finned fish, and mammals [83,84]. In the latter, it is specifically expressed only in the brain. This pattern of expression has led to the hypothesis that this isoform can function only in more evolved brains, with an implication in the regulation of food intake [82,85].

On the contrary, *D. melanogaster* has a single *CPT1* gene that shares a higher similarity with the human isoform A than with the other variants [86]. This gene is called *withered* (*whd*) and is referred to in FlyBase by the symbol *DmelCG1289*. *DmelCPT1/whd* encodes, through an alternative splicing, two different isoforms of the CPT1 protein [82]. As mammals, these isoforms differ in their activation and inhibition kinetics and are differentially expressed in flight muscle and fat body. Specifically, the isoform, predominantly expressed in flight muscle, as expected for tissue with high levels of fat oxidation, had overall greater enzymatic activity in comparison with the fat-body isoform. *dmCPT1/whd* mutants exhibit a crinkled wing phenotype, increased lipid storage, and are highly sensitive to starvation and oxidative stress induced by paraquat, heavy metals, and ethanol [87] due to a disruption of FA metabolism. Mutations of human CPT1 also lead to an increase of free fatty acid levels, great sensitivity to fasting throughout life, and increased ROS production due to the inability to import LCFA inside the mitochondria to generate ATP through their breakdown [88,89,90].

#### 3.1.2. Carnitine Acyl-Carnitine Translocase

In the second step of the CS, CACT, located in the inner mitochondrial membrane, allows acylcarnitine to cross the inner mitochondrial membrane by exchanging it with a free L-carnitine/acetylcarnitine molecule from the mitochondrial matrix [91,92]. In Drosophila, its homologous gene is called *congested-like trachea (colt)* and is referred to in FlyBase by the symbol *DmelCG3057*. Besides a high degree of amino-acidic similarity (71%) shared between human and Drosophila CACT, Oey et al. [93] showed that Drosophila COLT works as a carnitine acyl-carnitine transporter since it was able to rescue a yeast CACT deletion strain. It is highly expressed in all developmental stages, such as early embryonic development and morphogenesis, when energy requirement is high and fatty acid oxidation is essential [94]. *colt* mutants show an impairment in wings morphogenesis and collapsed tracheal trees due to a failure of energy production that feeds the transport of liquid through the tracheal epithelium, as initially postulated by Hartenstein et al. [94] and confirmed by Oey et al. [93]. Furthermore, *colt* mutants suggest that this protein, besides having indispensable roles for normal embryonic development, is important for larval survival and female fertility, resulting in severely affected colt-/- flies [94]. Human *CACT* deficiency causes neonatal or infantile sudden death suggesting, also in humans, the essential role of this gene in early embryonic development. In most patients, clinical features include neurologic abnormalities (lethargy, poor feeding), cardiac rhythm disorders, skeletal muscle damage, and liver dysfunction.

#### 3.1.3. Carnitine PalmitoylTransferase 2

CPT2, located at the mitochondrial inner membrane, catalyzes the third and last step of the CS. The Drosophila orthologous gene *CPT2* is referred to in FlyBase by the symbol *Dmel\CPT2* (*CG2107*). The Dmel\CPT2 enzyme shares a 67% amino acid sequence similarity with the human protein and is expressed throughout the body. Null mutants for *CPT2* result in early adult lethality, a severely reduced adult lifespan, and high sensitivity to starvation, pointing out the need for β-oxidation for adult fly survival. Furthermore, CPT2 deficiency leads to an interesting accumulation of triacylglyceride-filled lipid droplets only in the glial cells of the adult brain and flight muscle [95]. The authors also reported that this condition could be rescued by inducing the expression of *CPT2* in glial cells alone and, based on these results, suggested that, in Drosophila, the adult brain was able to utilize LCFA as fuel for cellular energy production.

In humans, *CPT2* deficiency causes sensitivity to fasting throughout life, early death, and brain defects [96,97]. However, although these brain defects and CPT2 expression in the adult brain have been clearly reported [98,99], to date, the role of CPT2 in the mammalian brain is still unclear and whether the mammalian brain can use β-oxidation for energy production remains a debate [100,101].

### 3.2. L-Carnitine and Peroxisomes

**Figure 3:**
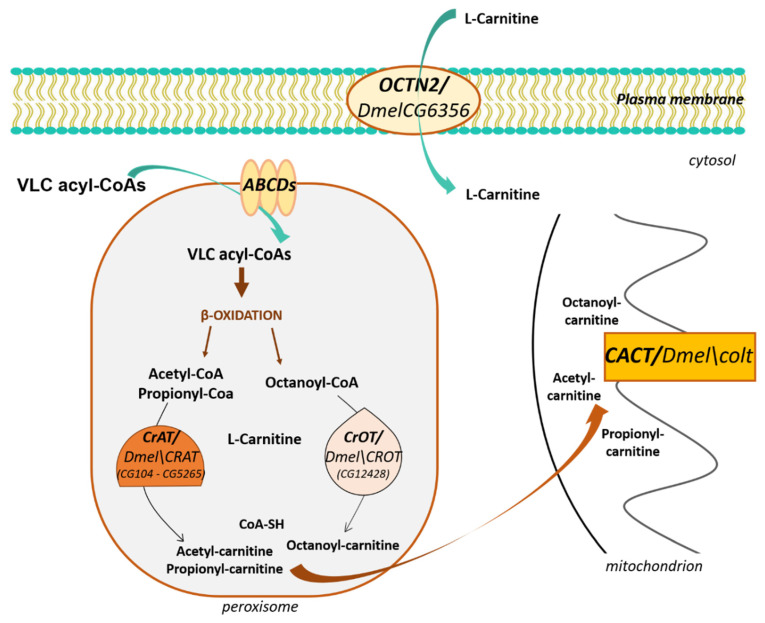
L-Carnitine and peroxisomes. The Drosophila orthologs are reported near each enzyme with the Dmel name or/and the corresponding FlyBase CG number(s).

β-oxidation of specific carboxylic acids such as VLCFA and branched-chain fatty acids, which cannot enter the mitochondria since they are not substrates of CPT-1, occurs in peroxisomes [72,73,74,102,103]. Their uptake is considered carnitine-independent and is mediated by ATP-binding cassette (ABC) transporters (ABCD1, ABCD2, and ABCD3) in the peroxisomal membrane [104]. However, L-carnitine also plays an essential role in peroxisomal β-oxidation. It is used by carnitine acyltransferases localized in the peroxisomal matrix to produce acylcarnitine esters of shortened fatty acids for transporting out of peroxisomes [10]. Precisely, peroxisomes work as a chain-shortening system of VLCFA or branched FA to generate acyl-CoAs of various chain lengths, including acetyl-CoA and propionyl-CoA. These products are converted into carnitine esters through a transferase reaction catalyzed by peroxisomal carnitine acetyltransferase (CrAT), whose substrates are short-chain acyl-CoAs and carnitine octanoyltransferase (CrOT), whose substrates are medium- and long-chain fatty acyl groups [8,103]. These carnitine esters are then transported out of peroxisomes, probably to mitochondria to complete β-oxidation, and generate additional energy (Figure 3) [73,74,75].

As vertebrates, Drosophila has the enzymes required for peroxisomal β- oxidation of VLCFAs, as suggested by mutant flies for the peroxins pex10 or pex16, which display elevated VLCFA levels and normal levels of shorter-chain fatty acids [105,106]. In Drosophila, the shortened products may be converted into carnitine esters through a transferase reaction catalyzed by the orthologs of carnitine acetyltransferase (CrAT) and Carnitine O-Acetyl-Transferase (CrOT). Faust et al. [107], by analyzing the proteome of Drosophila, identified two putative orthologs for CrAT and one for CrOT.

#### CrAT and CrOT

Two putative paralogs of CrAT are referred to in FlyBase by the symbols *Dmel\CRAT (CG104)* and *Dmel\CRAT (CG5265),* respectively. Both are still uncharacterized genes and their encoded proteins share a 58% similarity with hCRAT. The *CrOT* gene is referred to in FlyBase by the symbol *Dmel\CROT (CG12428)* and is the putative ortholog of human CROT. The encoded protein shows a 50% similarity with hCROT and remains uncharacterized yet. Significantly, all these proteins terminate in a peroxisome targeting signal 1 (PTS1) variant (-KNPPETKSKL), chosen by Faust et al. as a prototypical peroxisomal consensus for expressing GFP markers inside the peroxisome [107].

## 4. L-Carnitine Transport through the Plasma Membranes

L-Carnitine is a water-soluble molecule and requires specific transporters to cross the plasma membranes. In humans and mice, the high-affinity carnitine transporter organic cation transporter novel 2 (OCTN2) plays an essential role in the cellular uptake, tissue distribution, and renal reabsorption of L-carnitine (Figure 3). It is encoded by the *SLC22A5* gene, the mutations of which cause primary carnitine deficiency, an autosomal recessive disease characterized by decreased concentrations of carnitine within the serum and cells [8]. Interestingly, the mouse Slc22a5 knockout line, also known as juvenile visceral steatosis (JVS) line, is the only SLC22 KO mouse line that, to date, shows a clear developmental phenotype with Octn2 KO mice that die in 3–4 weeks after birth [108].

OCTN2 belongs to the SLC22 (Solute Carrier) family of transporters. Based on the nature of the substrate transported, this family has been subdivided into three subfamilies: organic anion transporters (OATs), organic cation transporters (OCTs), and organic zwitterion/cation transporters (OCTNs). Phylogenetic analysis of SLC22 members suggests the presence of 25 putative orthologs in flies, with no direct orthologs for OCTN2 [109]. However, the functional characterization of three SLC22 Drosophila orthologs, BalaT, CarT, and SLC22A [110,111,112,113], indicates that Drosophila SLC22 proteins can share substrates and functions with their human SLC22 counterparts. To obtain more information on the SLC22 fly members, Engelhart et al. [114] recently classified them utilizing multiple-sequence alignments and specific RNAi knockdowns. Interestingly, the authors reported that the Drosophila protein CG6356 not only shares a distinct homology with SLC22A16 (identity 28%, similarity 46%), but the RNAi knockdown of its gene was lethal at the pupa stage, highlighting the relevance of this gene on development. SLC22A16 (CT2) is a high-affinity carnitine transporter related to OCTNs, specifically expressed in human testis, where it is involved in the maturation of human spermatozoa by mediating L-carnitine secretion from the epididymal epithelium of the testis into the lumen [115]. In Drosophila, *CG6356* expression is not limited to the testis, as suggested by FlyAtlas [116], which reports *CG6356* expression in several tissues, such as CNS and fat body (http://flyatlas.org/atlas.cgi). Based on the established L-carnitine transport of SLC22A16 and the implications of *CG6356* knockdown on development, it has been suggested that the pupal lethality may be due to a systemic imbalance of L-carnitine [114,117], as previously reported for Octn2 (Slc22a5) KO in mice. Further investigation of CG6356 will be necessary to confirm that this gene might function as OCTN2, which could help to better understand the role of L-carnitine in the Drosophila nervous system.

## 5. L-Carnitine Antioxidant Properties

**Figure 4:**
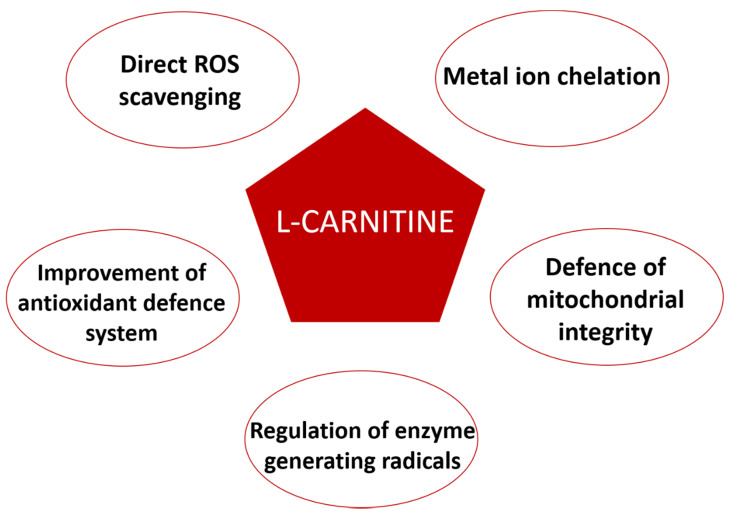
An overview of the antioxidant properties of L-carnitine.

The antioxidant action of L-carnitine is related to its involvement in protecting the cell against oxidative stress (Figure 4). L-Carnitine has a direct radical oxygen species (ROS) scavenging property, probably given by a carboxylate group with a carbonyl unit that stabilizes the radical formed [118,119]; in fact, the L-carnitine molecule is able to scavenge the 1,1-diphenyl-2-picryl-hydrazyl free radical, superoxide anion, and hydrogen peroxide [118,120].

In addition to its scavenging ability, the L-carnitine antioxidant effect is also due to its capability to regulate the enzymes generating free radicals, such as NADPH oxidase, an oxidoreductase producing ROS by transferring electrons from NADPH to molecular oxygen. It has been reported that this enzyme is indirectly modulated by L-carnitine through a dose-dependent inhibition of the Protein Hinase C (PKC), which in turn changes NADPH to a major cytosolic component phosphorylation state [121,122].

To counteract the oxidative stress, the cell presents a robust antioxidant defense system consisting of SuperOxide Dismutase (SOD), Glutathione Peroxidase (GPx), and Catalase (CAT). SOD converts anion superoxide by its dismutation to hydrogen peroxide and water, whereas CAT and GPx catalyze the decomposition of hydrogen peroxide into water, inactivating the toxic radicals. L-Carnitine may improve the activity of these enzymes [123]. Cao and colleagues reported an increase in the plasmatic concentration of these enzymes after a single-dose administration in healthy subjects. Furthermore, there is evidence about its involvement in the activation of pathways against oxidative stress, such as PPAR-α and NRF_2_, whose expressions result increased by L-carnitine treatment in hepatocytes, with the consequent activation of SOD and CAT [124,125,126].

One of the main targets of oxidative damage in the cell is the mitochondrion, which can be damaged. ROS can induce damages to the mitochondrial DNA, the mitochondrial respiratory chain, the permeability of mitochondrial membranes, and can alter the calcium homeostasis [127]. L-Carnitine shows protective effects of both mitochondrial structure and function, as reported by Kumaran and colleagues [128], showing the improvement of the electron transport chain enzymes after the L-carnitine treatment. Furthermore, L-carnitine effects on PPAR-α and NRF_2_ pathways and SOD and CAT activation also result in protective action on mitochondria [126].

In addition, the capability of L-carnitine to chelate the catalytic form of ROS reaction promoting metals, such as copper and iron, and to avoid their involvement in ROS formation through Fenton reaction should not *be* overlooked. The biochemical mechanism is based on the generation of a complex between L-carnitine hydroxyl and carboxylate groups and the metal ion, resulting in a chelation power comparable to EDTA [118,129].

Despite the generation of several Drosophila models to study oxidative stress [130,131], none of these shed light on the direct involvement of the L-carnitine in defense mechanisms against oxidative damage. Oxidative stress in Drosophila induces a shorter lifespan, locomotory defects, decreased activity of SOD, CAT, and GPx, and increased levels of ROS [132,133]; therefore, the effects on these phenotypes obtained by the genetic and/or pharmacological manipulation of the carnitine shuttle components and lipid oxidation pathway could suggest the correlation between the free-radical damage effects and L-carnitine [39].

## 6. L-Carnitine in Drosophila Brain Physiology

The transport of LCFA into the mitochondria performed by the CS represents a crucial step for the mitochondrial β-oxidation of FAs [134]. This is mainly seen in patients with inherited disorders such as hypoketotic hypoglycemia, cardiomyopathy, skeletal myopathy, and rhabdomyolysis. These genetic diseases can be caused by mutations in genes such as CPT1, CACT, and CPT2 [135,136], leading to defective FA oxidation. In these cases, when fat is released from adipose tissue, such as during fasting, defective fatty acid oxidation does not allow its utilization as fuel, which leads to an abnormal accumulation in skeletal muscle, heart, and liver. Although many of the diseases caused by mutations in these genes have been already characterized, in the last decade, it has been shown that the regulation of CS components is more complex than previously anticipated. Its dysfunction has a role in the brain physiology and plays an important part in the determination of organismal longevity.

It is commonly known that the adult brain utilizes glucose or, in fasting conditions, ketone bodies to generate ATP [137,138]. The utilization of FAs by the brain to produce energy has remained uncertain [139,140], although several papers support its importance for the brain [141,142]. CPT1, CPT2, and the carnitine transporter SLC22A5/OCTN2 are expressed in several regions of rat brain; in addition, a deletion in the *OCTN2* gene has been associated with attention-deficit/hyperactivity disorder [32], and mutations in the HTML gene were considered to be a risk factor in autism [34]. Taken together, these studies might suggest an essential role of L-carnitine for the physiological functioning of the brain.

By using a combination of metabolomic and transcriptomic profiling, Manzo et al. [36] identified an accumulation of carnitine conjugated LCFA in a Drosophila model of Amyotrophic Lateral Sclerosis (ALS) caused by a possible defect in the CS. This model, expressing the human TAR DNA binding protein 43 (TDP-43) in neuron and glial cells, showed locomotor dysfunction, a decreased lipid β-oxidation, and an altered transcriptional profile of the carnitine shuttle genes. Interestingly, feeding flies with medium-chain fatty acid (MCFA) that differently from LCFA can freely cross the mitochondrial membranes and partially rescue the locomotor defect associated with the pathology [36]. In a Drosophila model of Huntington’s disease (HD), expressing the mutated Huntingtin (Q128HD-FL) in the fly nervous system, Di Cristo et al. [38,39] reported that the pharmacological inhibition of the carnitine system led to a rescue of the disease phenotypes, such as locomotion defects and lifespan. Supporting this, Di Cristo et al. also showed a reduction of the protein aggregates in mouse STHdhQ111/111 striatal neuronal cells. These data are in accordance with the hypothesis that in HD models, a dysregulated carnitine cycle increases the FAO, leading to an accumulation of lipid metabolic intermediates and ROS as well as a decrease of glucose utilization. Age-related diseases are often characterized by the functional decline of different cellular processes needed to maintain organismal physiology.

A marked decline with age of the main enzymes of the CS, FAO, and carnitine metabolites has been seen to be a common characteristic in Drosophila, mouse, and human models [143,144,145]. Interestingly, ubiquitous overexpression throughout the body of genes related to FAO can extend longevity in flies and enhance tolerance to both starvation and oxidative stress [146]. These effects resemble calorie restriction that elicits, via AMPK (5′ AMP-activated protein kinase or *AMPK*), the activation of FA β-oxidation genes to produce energy from fat [147,148]. However, lifespan can be considered just a parameter of aging and does not take into account the relation between metabolic regulation and the functional decline of specific organs over time. Therefore, to study the aging process in a tissue-dependent manner, Laranjeira et al. [37] reported that in Drosophila brains, the upregulation of genes involved in FA β-oxidation had a critical role in age-related functional decline. Furthermore, the authors described a rescue of the age-related functional decline of the brain following the neuronal downregulation of some genes as well as *CG10814*, the ortholog for the hγ-BBH and DmelHNF4 (hepatocyte nuclear factor 4), the receptor that is supposed to work like PPAR-α in vertebrates [149]. This gene can sense free LCFAs levels derived from triglycerides hydroxylation upon nutrient limitation and elicits the activation, among others, of the *Dmel/CPT1 whd* gene, involved in importing acyl into mitochondria, and *Dmel/CG10814,* the *γ-BBH* ortholog encoding for the enzyme that catalyzes the last step of the carnitine biosynthesis pathway [150].

To challenge the dogma that β-oxidation of FAs is completely absent from the brain and highlight the importance of this pathway for brain physiology, Schulz et al. [95] used a CPT2 mutant Drosophila model (*Dmel/CPT2mut*). *Dmel/CPT2mut* flies die a few days after eclosion, showing the same dramatic phenotype of human patients affected by CPT2 deficiency, offering a powerful model system to study this disease. *Dmel/CPT2mut* accumulate triacylglycerides (TAG) in lipid droplets present in glia, and the glial-specific expression of the CPT2 human isoform is able to rescue the TAG level, demonstrating that glial cells can perform β-oxidation; however, to date, it remains unknown in which specific subtypes of glial cells β-oxidation occurs [95]. Further, the glial-specific expression of the CPT2 human isoform rescued lethality and lifespan probably due to a release of ketone bodies back into the hemolymph from glial cells [151].

## 7. Conclusions

L-Carnitine is an important molecule that plays a key role in the metabolism of fatty acids, enabling the shuttling of long-chain fatty acyl CoA to fuel mitochondrial β-oxidation, as well as in the maintenance of cellular energy homeostasis. Emerging studies suggest that L-carnitine plays an essential role in the control of brain functions. The use of a model organism could help to identify and characterize the physiological pathways in neurodegeneration. Drosophila remains an excellent model in unraveling many neurodegenerative disease mechanisms as well as identifying potential therapeutic targets. Therefore, studying the L-carnitine system in fruit flies could allow obtaining evidence of the pathogenic role played by the dysregulated carnitine cycle in models of neurodegenerative diseases to screen for drugs. This review summarizes the current knowledge on L-carnitine in Drosophila, emphasizing its strong genetic and biochemical conservation with humans, and discusses the role of the L-carnitine system in fly models of neurodegeneration. In conclusion, all these reports offer direct or more indirect evidence that a dysregulated L-carnitine cycle can lead to an increase of FAO in the brain, with an accumulation of lipid metabolic intermediates and ROS, highlighting the power of the fruit fly in unraveling the mechanism underlining these disorders. However, further studies should be undertaken to further understand the role of L-carnitine on brain physiology and identify novel therapeutic targets for neurodegeneration.
